# The power of sound: unravelling how acoustic communication shapes group dynamics

**DOI:** 10.1098/rstb.2023.0182

**Published:** 2024-06-23

**Authors:** Elodie F. Briefer, Bing Xie, Sabrina Engesser, Cedric Sueur, Todd M. Freeberg, Josefine Bohr Brask

**Affiliations:** ^1^ Behavioural Ecology Group, Section for Ecology & Evolution, Department of Biology, University of Copenhagen, Copenhagen 2100, Denmark; ^2^ Institut Pluridisciplinaire Hubert Curien, Université de Strasbourg, CNRS, UMR 7178, Strasbourg 67087, France; ^3^ Department of Psychology and Department of Ecology and Evolutionary Biology, University of Tennessee, Knoxville, TN 37996, USA

**Keywords:** bioacoustics, collective behaviour, social networks, social interactions, vocal communication, animal sociality

## Abstract

Acoustic signalling is a key mode of communication owing to its instantaneousness and rapid turnover, its saliency and flexibility and its ability to function strategically in both short- and long-range contexts. Acoustic communication is closely intertwined with both collective behaviour and social network structure, as it can facilitate the coordination of collective decisions and behaviour, and play an important role in establishing, maintaining and modifying social relationships. These research topics have each been studied separately and represent three well-established research areas. Yet, despite the close connection of acoustic communication with collective behaviour and social networks in natural systems, only few studies have focused on their interaction. The aim of this theme issue is therefore to build a foundation for understanding how acoustic communication is linked to collective behaviour, on the one hand, and social network structure on the other, in non-human animals. Through the building of such a foundation, our hope is that new questions in new avenues of research will arise. Understanding the links between acoustic communication and social behaviour seems crucial for gaining a comprehensive understanding of sociality and social evolution.

This article is part of the theme issue ‘The power of sound: unravelling how acoustic communication shapes group dynamics’.

## 1. Introduction

In many species, acoustic communication is essential to coordinate social activities and to build and maintain stable and sustainable social structures. Acoustic signals can encode many pieces of information about the emitter, including both static (e.g. species, sex, individuality) and dynamic (e.g. motivation, emotion) information, and can in some species also carry information about external events (e.g. presence and type of predator, food) [1]. This information is often perceived and used by receivers, hence influencing their behaviour and subsequent social interactions. For instance, variation in acoustic signals can stem from variation in the emotional/affective state of the signaller (e.g. in horse calls, *Equus caballus* [2] and *Equus przewalskii* [3]), and this acoustic variation can influence the behaviour of receivers [4,5]. In the context of collective group behaviour, many group-foraging species produce contact calls to coordinate group movements [6]. For example, the short-range ‘close’ calls produced by meerkats (*Suricata suricatta*) during foraging are affected by the spatial distance and alignment to neighbouring group members, and these calls elicit call responses and approaches by receivers, facilitating group cohesion during movement [7]. Besides coordinating collective behaviours, vocalizations can also encode information about the social relationships within a group. For example, in bottlenose dolphins (*Tirsiops aduncus*), individual vocal labels serve to recognise cooperative partners or competitors in multi-level alliances [8]. In Carolina chickadees (*Poecile carolinensis*), individuals placed into captive aviary flocks that are unfamiliar with one another call differently from individuals placed into captive aviary flocks that are familiar with one another (the former flocks were captured from different wild flocks and the latter from the same wild flocks [9]). Thus, acoustic signals can encode diverse types of information and can affect—and be affected by—many aspects of social behaviour. They are therefore closely connected to both collective behaviour and social structuring.

Collective behaviour has gained a lot of scientific interest in recent years and has been studied in many species [10,11]. Collective behaviour requires coordination between individuals, and acoustic signalling provides an excellent means to facilitate this [12]. It has also long been known that animals use acoustic signals for maintaining group cohesion during movement (e.g. [13,14]) and that acoustic communication is therefore important for collective behaviour. However, the complex and diverse ways in which acoustic signals are used in collective behaviour are only starting to be revealed. Studying the connection between acoustic communication and collective behaviour is important for gaining a comprehensive understanding of social systems [12].

Similarly to collective behaviour, social network structures have by now been quantified and studied widely across animal taxa, and they have been shown to play an important part in processes such as disease and information transmission [15], and for the fitness and well-being of individuals [16]. It is therefore important to understand how these structures emerge and are maintained. Given the important role that acoustic signals play in coordinating collective activities and social interactions, it follows that these signals are likely to have a close relationship with social structuring in many species; they can be used to establish and modify social relationships (which together form the social structure), and the social structure can, in turn, affect acoustic communication—both on short timescales and evolutionarily. For example, one aspect that has already received considerable attention is the relationship between vocal and social complexity: the diversity of acoustic signals (i.e. variation within and across signals) may be driven largely by the complexity of the social structure experienced by individuals [17–20], and variation in acoustic signals might in turn influence social interactions and relationships in ways that drive changes in social structure [21].

The study of how acoustic signals interact with social dynamics in non-human animals is a powerful—but rarely used—way to understand the evolution of non-dyadic vocal communication and its relationship to individual decision-making processes and social cognition [22–24]. It is also important for understanding how animals engage in both prosocial and competitive interactions and relationships, and for gaining insights into the development and maintenance of societies both in non-human animals and humans. Through vocal signals, individuals influence the formation and dynamics of social networks. These networks exhibit characteristics like modularity and polarity, where certain individuals hold more influence or connections [25,26]. Importantly, feedback mechanisms exist where acoustic signals and the ensuing social interactions influence voting and decision-making processes within the group. Simultaneously, collective choices impact the social network structure, creating a continuous feedback loop [27]. This interplay between acoustic communication, collective behaviour and social networks forms a dynamic system where the voices of individuals and the bonds they form interact with decision-making processes.

Given the decades of study aimed at understanding each of the three focal areas of this theme issue separately (acoustic communication, collective behaviour and social networks) and an increasing interest in merging them (e.g. [12,28–30]), we are now in a strong position to expand the integration of the three areas. As mentioned above, the study of collective behaviour and of social networks is now well established and is currently undergoing exciting expansions and transformations driven by newly developed analytical methods and state-of-the-art tools. These two areas of study have, however, only rarely been integrated with acoustic communication. Some more recent work indicates that such integration can be done and can be deeply revealing. For example, both visual and acoustic signals and cues are used by individual zebra finches (*Taeniopygia guttata*) in coordinating flock flight, and when visual information is diminished or blocked, individuals rely much more on acoustic information to avoid collisions with other individuals [31]. Male bottlenose dolphins that have functionally important but relatively weak social bonds with one another rely heavily on vocal signalling in ways that maintain those bonds [29]. Similarly, although strong social relationships in chimpanzees (*Pan troglodytes*) are maintained largely through visual signals and visual/vocal multimodal signal combinations, weaker relationships among individuals are maintained by vocal signals [32]. For chimpanzees, furthermore, greeting calls are used in instances when smaller groups come back together in fusion, and the characteristics of these calls predict subsequent agonistic interactions and physical proximity of signallers and receivers [33]. Finally, calling intensity of jackdaws (*Corvus monedula*) increases steadily in the tens of minutes prior to morning departure from their roosts, and playbacks of jackdaw vocalizations result in earlier departure from roosts [30]. These recent publications are a few examples of an exciting increase in interest in the role that acoustic communication plays in collective behaviour and social network structures. This theme issue includes 15 papers that consider the connection between acoustic communication and social dynamics, and we hope that this can trigger the expansion of this exciting and important research area.

## 2. Overview of the theme issue

This theme issue comprises an opening theory/review article and two main sections. In the opening paper, Xie *et al*. [34] review the findings of studies that reveal the importance of acoustic signals to collective movement, group cohesion maintenance and separation risk mitigation, fission–fusion dynamics and the structuring of social networks. The authors stress the need for work on a wider range of species and systems (particularly beyond Primates and Passeriformes) and the strengths of recent technology to aid in answering questions related to communication and group-level social dynamics. We provide an overview of the papers in the two main sections of the theme issue below. The first main section is composed of articles that integrate acoustic communication and collective behaviour, and the second main section contains articles that integrate acoustic communication and social networks (or general group characteristics).

### (a) Acoustic communication and collective behaviour

In the first paper on the link between acoustic communication and collective behaviour, Liao *et al*. [35] emphasize the utilization of acoustic signals and movement cues in collective decision-making processes. This paper provides an overview of how these signals or cues have been employed to coordinate collective behaviours and introduces a conceptual framework to discern which types of collective behaviours rely on acoustic signals or movement cues. By outlining information masking, discrimination ability (Weber’s law) and encoding limitations, as well as trade-offs between these, the authors propose that acoustic signals are typically used in behaviours that involve temporal coordination or the expression of specific actions, whereas movement cues are more commonly employed in decisions that entail complex choices with multiple options, such as direction or destination selection. Furthermore, the authors suggest avenues for future research, including exploring multimodal communication and collective decision-making in mixed-species animal groups.

Bousquet *et al*. [36] provide a framework to investigate the role that ecological and social heterogeneity may play in the evolution of complex communication. By reviewing vertebrate decision-making processes, the authors discuss different types of information transfer that animals use in three key behavioural contexts: transitioning between activities, foraging coordination and group movements. Under the assumption of a positive correlation between ecological and social heterogeneity, Bousquet *et al*. [36] postulate that different magnitudes of heterogeneity should select for communication of differing complexity with respect to how information is conveyed (i.e. inadvertent cues versus evolved signals) and integrated (i.e. copying versus negotiation behaviour). Specifically, low heterogeneity is hypothesized to select for information transfer via simple copying of inadvertent cues, while high heterogeneity is hypothesized to select for information transfer via more complex integration mechanisms and complex signals. Bousquet *et al*. [36] further discuss the use of new technologies to study communication dynamics and to test the proposed link between the complexity of a communication system and social and ecological heterogeneity, within and across species.

Broad *et al*. [37] compare rural and urban sites in southwestern England to assess whether wild jackdaw groups exhibit different roosting, collective movement and vocal behaviour during the night-time, depending on levels of anthropogenic noise. The authors collected audio and video recordings from several jackdaw roosts at five different locations—one very urban, a second moderately urban and three relatively rural. Compared to jackdaws from rural areas, birds from urban areas with higher levels of traffic noise take longer to settle in the roost once they have arrived and they call more during the night. Urban birds also exhibit less coordinated group departures from the roosts during the mornings. These findings suggest that high levels of anthropogenic noise (acoustic and light pollution) negatively impact attention and perception, resulting in the disruption of collective movement and sleep in these highly social corvids.

Sagot *et al*. [38] investigate the impact of the number of informed individuals and signal reliability on group cohesion and consensus among Spix’s disk-winged bats (*Thyroptera tricolor)*. The authors explored whether the number of roost broadcasting calls affects group fragmentation and whether calling rates influence the consensus on roosting choices. The findings reveal a positive correlation between the number of roost broadcasting calls and the likelihood of group fragmentation across multiple roosts. Additionally, a majority of group members enter roosts with higher calling rates, suggesting a role in reducing the risk of fragmentation. The authors underscore the potential drawbacks of having an abundance of information producers for group coordination, despite their importance in locating vital resources.

Demartsev *et al*. [39] highlight the nuanced roles of acoustic communication in meerkats, focusing on how ‘close calls’ and ‘short note calls’ serve distinct functions within the social structure of this species. Close calls, essential for maintaining group cohesion, follow a ‘call and response’ pattern with high caller exchange probability, influenced by the presence and age class of nearby conspecifics. In contrast, short note calls, not influenced by the number of nearby conspecifics, are broadcast signals reflecting the individual’s response to external events or changes in behavioural state, particularly during rapid movement. These findings underscore the complexity of acoustic communication in facilitating social cohesion and navigation, demonstrating that the temporal structure of vocal signals provides critical insights into their functions and the social dynamics of meerkat groups.

Longing *et al*. [40] highlight the role of wild zebra finches (*Taeniogypia castanosis*) as a model songbird in investigating song systems, and suggest that researchers might have developed a less dualistic view of bird song functions if research had initially focused on wild zebra finches. Zebra finches live in less predictable and highly fluctuating environments compared to the extensively studied territorial species in the Northern hemisphere that have thereby become the foundation of our birdsong systems understanding. In contrast to these species, which sing in classical contexts of mate choice and territoriality, zebra finches primarily sing all year round in a range of social contexts. By reviewing research on zebra finches in both the lab and in the wild, the authors summarize the function of their songs as individual signatures, and as within-group signals, considering their low amplitude. Moreover, they also predict that zebra finches have evolved other mechanisms to regulate spatial relationships, likely including the integration of vocal signals and spatial routines, and utilizing water sources and social hotspots (trees or bushes that they gather around) as gathering points. In conclusion, the authors provide an overview of how zebra finches’ songs can facilitate social cohesion and coordination in short spatial ranges. They also encourage further studies on social hotspots and the evolution of the low-amplitude song to explore the extent to which wild zebra finches can be a representative species in understanding birdsong systems.

### (b) Acoustic communication and social networks (or group characteristics)

In the first paper on the link between acoustic communication and social network structure, Iacopini *et al*. [41] explore the significance of higher-order networks in studying animal vocal communication, moving beyond traditional dyadic interactions to understand complex, non-dyadic social structures and their impact on vocal behaviours. This paper presents three case studies demonstrating the advantages of higher-order network models in analysing vocally coordinated group departures, song synchronization and the cultural evolution of vocal communication. These models offer fresh insights and alter traditional predictions, underscoring their potential to address behavioural, ecological and evolutionary questions that were previously challenging to explore. The article stresses the value of interdisciplinary collaboration in pushing forward the boundaries of network science and biology, particularly in incorporating individual heterogeneity and temporal dynamics into models. It highlights the necessity for advanced data collection and computational methods to manage the complexities introduced by temporally dynamic higher-order networks. Ultimately, this discussion emphasizes how higher-order network approaches can revolutionize our understanding of animal vocal communication, inspiring new theoretical models and methodological advancements while tackling intricate research questions about social interaction patterns among animals.

Reichert *et al*. [42] investigate the impact of variation in characteristics of both signallers and receivers such as chorus size, signal perception, variation in individual call loudness and duration, and spatial position, on the properties of a communication network (including connection strength and centrality) and the resulting feedback on individual competitive interactions. Using simulations based on acoustic chorusing behaviour in lek-breeding treefrogs, the authors found that individual characteristics of both signallers and receivers have an impact on the structure of the communication network, which, in turn, influences individual competitive interactions. Overall, this paper relates important predictions for the study of communication networks, which could be investigated experimentally in the future.

Langehennig-Peristenidou and Scheumann [43] assess relationships between social grouping and changes in the acoustic features of trills of captive mouse lemurs (*Microcebus murinus)*. In this species, females regularly form sleeping groups, whereas males are solitary most of the time. The authors discovered that the trill calls of individual females become more acoustically similar the more time they spend together in close social contact. By contrast, the trills of males that socialize regularly with one another become more dissimilar over time. Genetic relatedness of individuals is associated with increased acoustic similarity of trills for males but not for females. Taken together, these results point to the importance of social accommodation in these mouse lemurs on the fine acoustic structure of one of their major vocal signals.

Chereskin *et al*. [44] investigate the impact of social and non-social factors on male Indo-Pacific bottlenose dolphin vocal communication during a cooperative act. More precisely, the authors studied the features of ‘pop’ vocalizations, which are used as a signal between allied males to coerce single oestrus females and herd them away from their home ranges. They tested the influence of first-order alliance fidelity, cumulative first-order alliance bond strength and distance from the consorted female’s home range on pop rate, bout duration and the proportion of synchronous pops. Their results reveal that, contrary to their prediction, social or geographic factors do not influence pop rate or bout duration. However, social bond strength predicts the proportion of synchronous pop trains. The authors conclude that social bond strength is an important predictor of pop use in this cooperative context, revealing the important link between social network measures and vocal communication in dolphins.

Chaverri *et al*. [45] study the use of contact calls in the disc-winged bat and how this relates to social relationships. Contact calls play an important role in maintaining social cohesion and coordinating behaviour in many species, including disc-winged bats. This species forms small groups that roost together in furled leaves. The leaves unfold after about one day, and groups must therefore frequently find a new roost site, during which process they use contact calls to maintain group cohesion. Using an experimental setting, Chaverri *et al*. [45] tested how contact calls between a roosting bat and a flying bat depend on their kinship and social association (quantified from natural co-roosting). They find that calling rates do not correlate with kinship or social association, suggesting that contact calls in this species may not indicate within-group social relationships and are used to maintain contact with the whole group.

Fichtel *et al*. [46] study the link between dominance style, in terms of despotism, and the existence and use of unidirectional submissive signals, which can function both to signal submission following aggression (immediate behavioural response) and also subordination or affiliative intent in peaceful contexts (long-term behavioural pattern). This ability to signal long-term behavioural patterns has been proposed to enable higher-quality relationships, and hence more robust dominance relationships, and to be more common in less despotic species where uncertainty in dominance is higher. The authors compared submissive calls emitted among dyads of ringed-tailed lemurs (*Lemur catta*), which live in large groups characterized by a strongly despotic dominance hierarchy, and dyads of Verreaux’s sifakas (*Propithecus verreauxi*), which live in small groups with a more relaxed dominance hierarchy. The results show that both species emit submissive calls with a dual function. Unlike the authors' prediction, submissive calls produced in peaceful contexts are more common in the more despotic ringed-tailed lemurs. In addition, in ringed-tailed lemurs, but not in Verreaux’s sifakas, more affiliative—and less aggressive—dyads emit more peaceful calls. This contradicts the pattern found in macaques and suggests that more studies in other group-living primate lineages should be conducted to fully understand the evolution and function of submissive signals.

Walsh *et al*. [47] study the link between vocal and social complexity. The Social Complexity Hypothesis for Communicative Complexity states that social and vocal complexity should coevolve, and higher vocal complexity should therefore be found in more complex social environments [17]. Walsh and colleagues investigated this hypothesis by studying call combinations (where calls are combined into longer sequences) in Western Australian magpies (*Gymnorhina tibicen dorsalis*). Using observations and audio recordings from free-living magpie groups, they investigated whether the use of call combinations is connected to the size and composition of the groups. They find that in larger groups, calling magpies give call combinations more frequently and in greater diversity, supporting the hypothesis that vocal combinatorial complexity is linked to social complexity.

Lin *et al*. [48] emphasize the underestimated potential for vocal and acoustic communication in non-avian reptiles, traditionally considered solitary with reliance on vision and olfaction for interactions. Recent findings suggest that acoustic communication in these reptiles may be more common than previously thought, indicating a higher communicative complexity and more social interactions than assumed. The review calls for more extensive research on acoustic behaviours across species and contexts, highlighting that vocalization can aid in conservation efforts and reveal social complexities. It is argued that understanding vocalization in reptiles could provide insights into the coevolution of sociality and communication in terrestrial vertebrates. The review also suggests exploring the Social Complexity Hypothesis for Communicative Complexity in reptiles, proposing that those living in larger groups may exhibit greater vocal complexity. Additionally, it points to the potential of vocal reptiles to demonstrate advanced cognitive abilities necessary for social interactions. Overall, the review advocates for a broader investigation into the acoustic communication of non-avian reptiles to uncover their true social complexity and cognitive capabilities, challenging the notion of their solitary nature and contributing to conservation and sensory ecology.

## 3. Concluding remarks

This collection of reviews and research papers on the understudied link between acoustic communication and the two areas of collective behaviour and social networks provides readers with not only a comprehensive overview of the topic but also a theoretical foundation and empirical examples of how these fields can be integrated. We believe that future work on these topics will provide novel insights into how communication mediates social relationships and behaviour at the individual level and how it shapes and interacts with the structure and functioning of social systems at the group level. We hope that this theme issue will serve as a motivator for further theoretical and empirical work to help solidify and build upon this integration.

## Data Availability

This article has no additional data.

## References

[1] Aubin T , Mathevon N . 2020 Coding strategies in vertebrate acoustic communication. Cham, Switzerland: Springer Nature.

[2] Briefer EF , Maigrot AL , Mandel R , Freymond SB , Bachmann I , Hillmann E . 2015 Segregation of information about emotional arousal and valence in horse whinnies. Sci. Rep. **4** , 9989. (10.1038/srep09989) 25897781 PMC4404681

[3] Maigrot A , Hillmann E , Anne C , Briefer EF . 2017 Vocal expression of emotional valence in Przewalski’s horses. Sci. Rep. **7** , 8779. (10.1038/s41598-017-09437-1) 28821880 PMC5562828

[4] Briefer EF , Mandel R , Maigrot AL , Briefer Freymond S , Bachmann I , Hillmann E . 2017 Perception of emotional valence in horse whinnies. Front. Zool. **14** , 8. (10.1186/s12983-017-0193-1) 28203263 PMC5303229

[5] Briefer EF . 2018 Vocal contagion of emotions in non-human animals. Proc. R. Soc. B **285** , 20172783. (10.1098/rspb.2017.2783) PMC5832712 29491174

[6] Fichtel C , Manser M . 2010 Vocal communication in social groups. In Animal behaviour: evolution and mechanisms (ed. P Kappeler ), pp. 29–54. Berlin, Heidelberg, Germany: Springer. (10.1007/978-3-642-02624-9)

[7] Engesser S , Manser MB . 2022 Collective close calling mediates group cohesion in foraging meerkats via spatially determined differences in call rates. Anim. Behav. **185** , 73–82. (10.1016/j.anbehav.2021.12.014)

[8] King SL , Friedman WR , Allen SJ , Gerber L , Jensen FH , Wittwer S , Connor RC , Krützen M . 2018 Bottlenose dolphins retain individual vocal labels in multi-level alliances. Curr. Biol. **28** , 1993–1999. (10.1016/j.cub.2018.05.013) 29887310

[9] Coppinger BA , Sanchez de-Launay A , Freeberg TM . 2018 Carolina chickadee (Poecile carolinensis) calling behavior in response to threats and in flight: flockmate familiarity matters. J. Comp. Psychol. **132** , 16–23. (10.1037/com0000090) 28956937

[10] Petit O , Bon R . 2010 Decision-making processes: the case of collective movements. Behav. Proc. **84** , 635–647. (10.1016/j.beproc.2010.04.009) 20435103

[11] Sueur C *et al* . 2011 Collective decision‐making and fission–fusion dynamics: a conceptual framework. Oikos **120** , 1608–1617. (10.1111/j.1600-0706.2011.19685.x)

[12] Demartsev V , Gersick AS , Jensen FH , Thomas M , Roch MA , Manser MB , Strandburg‐Peshkin A . 2023 Signalling in groups: new tools for the integration of animal communication and collective movement. Meth. Ecol. Evol. **14** , 1852–1863. (10.1111/2041-210X.13939)

[13] Bousquet CAH , Sumpter DJT , Manser MB . 2011 Moving calls: a vocal mechanism underlying quorum decisions in cohesive groups. Proc. R. Soc. B **278** , 1482–1488. (10.1098/rspb.2010.1739) PMC3081741 21047853

[14] Walker RH , King AJ , McNutt JW , Jordan NR . 2017 Sneeze to leave: African wild dogs (Lycaon pictus) use variable quorum thresholds facilitated by sneezes in collective decisions. Proc. R. Soc. B **284** , 20170347. (10.1098/rspb.2017.0347) PMC5597819 28878054

[15] Evans JC , Silk MJ , Boogert NJ , Hodgson DJ . 2020 Infected or informed? Social structure and the simultaneous transmission of information and infectious disease. Oikos **129** , 1271–1288. (10.1111/oik.07148)

[16] Snyder-Mackler N *et al* . 2020 Social determinants of health and survival in humans and other animals. Science **368** , eaax9553. (10.1126/science.aax9553) 32439765 PMC7398600

[17] Freeberg TM , Dunbar RIM , Ord TJ . 2012 Social complexity as a proximate and ultimate factor in communicative complexity. Phil. Trans. R. Soc. B **367** , 1785–1801. (10.1098/rstb.2011.0213) 22641818 PMC3367695

[18] Pollard KA , Blumstein DT . 2012 Evolving communicative complexity: insights from rodents and beyond. Phil. Trans. R. Soc. B **367** , 1869–1878. (10.1098/rstb.2011.0221) 22641825 PMC3367702

[19] Peckre L , Kappeler PM , Fichtel C . 2019 Clarifying and expanding the social complexity hypothesis for communicative complexity. Behav. Ecol. Sociobiol. **73** , 1–19. (10.1007/s00265-018-2605-4)

[20] delBarco-Trillo J , Sacha CR , Dubay GR , Drea CM . 2012 Eulemur, me lemur: the evolution of scent-signal complexity in a primate clade. Phil. Trans. R. Soc. B **367** , 1909–1922. (10.1098/rstb.2011.0225) 22641829 PMC3367706

[21] McComb K , Semple S . 2005 Coevolution of vocal communication and sociality in primates. Biol. Lett. **1** , 381–385. (10.1098/rsbl.2005.0366) 17148212 PMC1626386

[22] Freeberg TM , Gentry KE , Sieving KE , Lucas JR . 2019 On understanding the nature and evolution of social cognition: a need for the study of communication. Anim. Behav. **155** , 279–286. (10.1016/j.anbehav.2019.04.014)

[23] Lucas JR , Gentry KE , Sieving KE , Freeberg TM . 2018 Communication as a fundamental part of Machiavellian intelligence. J. Comp. Psychol. **132** , 442. (10.1037/com0000138) 30451528

[24] Cheney DL , Seyfarth RM . 2019 Baboon metaphysics: the evolution of a social mind. Chicago, USA: University of Chicago Press.

[25] Matakos A , Terzi E , Tsaparas P . 2017 Measuring and moderating opinion polarization in social networks. Data Min. Knowl. Disc. **31** , 1480–1505. (10.1007/s10618-017-0527-9)

[26] Grover P , Kar AK , Dwivedi YK , Janssen M . 2019 Polarization and acculturation in US election 2016 outcomes – can twitter analytics predict changes in voting preferences. Technol. Forecast. Soc. Change **145** , 438–460. (10.1016/j.techfore.2018.09.009)

[27] Almaatouq A , Noriega-Campero A , Alotaibi A , Krafft PM , Moussaid M , Pentland A . 2020 Adaptive social networks promote the wisdom of crowds. Proc. Natl Acad. Sci. USA **117** , 11379–11386. (10.1073/pnas.1917687117) 32393632 PMC7260971

[28] Snijders L , Naguib M . 2017 Communication in animal social networks: a missing link? In Advances in the study of behavior (eds M Naguib J Podos , LW Simmons , L Barrett , SD Healy , M Zuk ), pp. 297–359, vol. 49. Amsterdam, The Netherlands: Elsevier. (10.1016/bs.asb.2017.02.004)

[29] Chereskin E , Connor RC , Friedman WR , Jensen FH , Allen SJ , Sørensen PM , Krützen M , King SL . 2022 Allied male dolphins use vocal exchanges to 'bond at a distance.' Curr. Biol. **32** , 1657–1663. (10.1016/j.cub.2022.02.019) 35334229

[30] Dibnah AJ , Herbert-Read JE , Boogert NJ , McIvor GE , Jolles JW , Thornton A . 2022 Vocally mediated consensus decisions govern mass departures from jackdaw roosts. Curr. Biol. **32** , R455–R456. (10.1016/j.cub.2022.04.032) 35609539

[31] Arnold F , Staniszewski MS , Pelzl L , Ramenda C , Gahr M , Hoffmann S . 2022 Vision and vocal communication guide three-dimensional spatial coordination of zebra finches during wind-tunnel flights. Nat. Ecol. Evol. **6** , 1221–1230. (10.1038/s41559-022-01800-4) 35773345 PMC9349042

[32] Damjanovic L , Roberts SGB , Roberts AI . 2022 Language as a tool for social bonding: evidence from wild chimpanzee gestural, vocal and bimodal signals. Phil. Trans. R. Soc. B **377** , 20210311. (10.1098/rstb.2021.0311) 35934964 PMC9358320

[33] Fedurek P , Tkaczynski PJ , Hobaiter C , Zuberbühler K , Wittig RM , Crockford C . 2021 The function of chimpanzee greeting calls is modulated by their acoustic variation. Anim. Behav. **174** , 279–289. (10.1016/j.anbehav.2021.02.002)

[34] Xie B , Brask J , Dabelsteen T , Briefer E . 2024 Exploring the role of vocalizations in regulating group dynamics. Phil. Trans. R. Soc. B **379** , 20230182. (10.1099/rstb.2023.0183) 38768197 PMC11391291

[35] Liao CC , Magrath R , Manser M . Farine D.2024 The relative contribution of acoustic signals versus movement cues in group coordination and collective decision-making. Phil. Trans. R. Soc. B **379** , 20230184. (10.1098/rstb.2023.0184) 38768199 PMC11391321

[36] Bousquet C , Sueur C , King A , O’Bryan L . 2024 Individual and ecological heterogeneity promote complex communication in social vertebrate group decisions. Phil. Trans. R. Soc. B **379** , 20230204. (10.1098/rstb.2023.0204) 38768211 PMC11391315

[37] Broad H , Dibnah A , Smith A , Thornton A . 2024 Anthropogenic disturbance affects calling and collective behaviour in corvid roosts. Phil. Trans. R. Soc. B **379** , 20230185. (10.1098/rstb.2023.0185) 38768208 PMC11391286

[38] Sagot M , Rose N , Chaverri G . 2024 Group vocal composition and decision-making during roost finding in Spix’s disk-winged bats. Phil. Trans. R. Soc B **379** , 20230187. (10.1098/rstb.2023.0187) 38768206 PMC11391296

[39] Demartsev V , Averly B , Johnson-Ulrich L , Sridhar V , Leonardos L , Thomas M , Vining A , Manser M , Strandburg-Peshkin A . 2024 Mapping vocal interactions in space and time differentiates signal broadcast vs signal exchange in meerkat groups. Phil. Trans. R. Soc. B **379** , 20230188. (10.1098/rstb.2023.0188) 38768207 PMC11391280

[40] Loning H , Griffith S , Naguib M . 2024 The ecology of zebra finch song and its implications for vocal communication in multi-level societies. Phil. Trans. R. Soc. B **379** , 20230191. (10.1098/rstb.2023.0191) 38768203 PMC11391294

[41] Iacopini I , Foote J , Fefferman N , Derryberry E , Silk M . 2024 Not your private tête-à-tête:leveraging the power of higher-order networks to study animal communication. Phil.Trans. R. Soc. B **379** . (10.1098/rstb.2023.0190) PMC11391305 38768202

[42] Reichert M , Luttbeg B , Hobson E . Collective signalling is shaped by feedbacks between signaller variation, receiver perception, and acoustic environment in a simulated communicatio nnetwork. Phil. Trans. R. Soc. B **379** , 20230186. (10.1098/rstb.2023.0186) PMC11391285 38768210

[43] Langehennig-Peristenidou A , Scheumann M . 2024 Sex differences in the impact of social relationships on individual vocal signatures in grey mouse lemurs (Microcebus murinus). Phil. Trans. R. Soc. B **379** , 20230193. (10.1098/rstb.2023.0193) 38768201 PMC11391318

[44] Chereskin E , Allen S , Connor R , Krützen M , King S . 2024 In pop pursuit: social bond strength predicts vocal synchrony during cooperative mate guarding in bottlenose dolphins. Phil. Trans. R. Soc B **379** , 20230194 (10.1098/rstb.2023.0194) 38768196 PMC11391284

[45] Chaverri G , *et al* . 2024 Calling to the collective: contact calling rates within groups of disc-winged bats do not vary by kinship or association. Phil. Trans. R. Soc. B **379** , 20230195. (10.1098/rstb.2023.0195) 38768198 PMC11391311

[46] Fichtel C , Dinter K , Ratsoavina FM . 2024 Benefits but not the dual function of submissive signals differ between two Malagasy primates. Phil. Trans. R. Soc. B **379** , 20230197. (10.1098/rstb.2023.0197) 38768209 PMC11391313

[47] Walsh S , Townsend S , Engesser S , Ridley A . 2024 Social environment influences call combination production in Western Australian magpies (Gymnorhina tibicendorsalis). Phil. Trans. R. Soc. B **379** , 20230198. (10.1098/rstb.2023.0198) 38768205 PMC11391283

[48] Lin FC , Lin SM , Godfrey S . 2024 Hidden social complexity behind vocal and acoustic communication in non-avian reptiles. Phil. Trans. R. Soc. B **379** , 20230200. (10.1098/rstb.2023.0200) 38768204 PMC11391309

